# Supplementary material for: Comparison of Conformations and Interactions with Nicotinic Acetylcholine Receptors for E. coli-Produced and Synthetic Three-Finger Protein SLURP-1
Source: Int J Mol Sci. 2023 Nov 29;24(23):16950. doi: 10.3390/ijms242316950 (PMC10707033; doi:10.3390/ijms242316950)
Supplement: Supplementary file 1 [file ijms-24-16950-s001.zip › ijms-2665111-supplementary.pdf]

Supplementary Materials

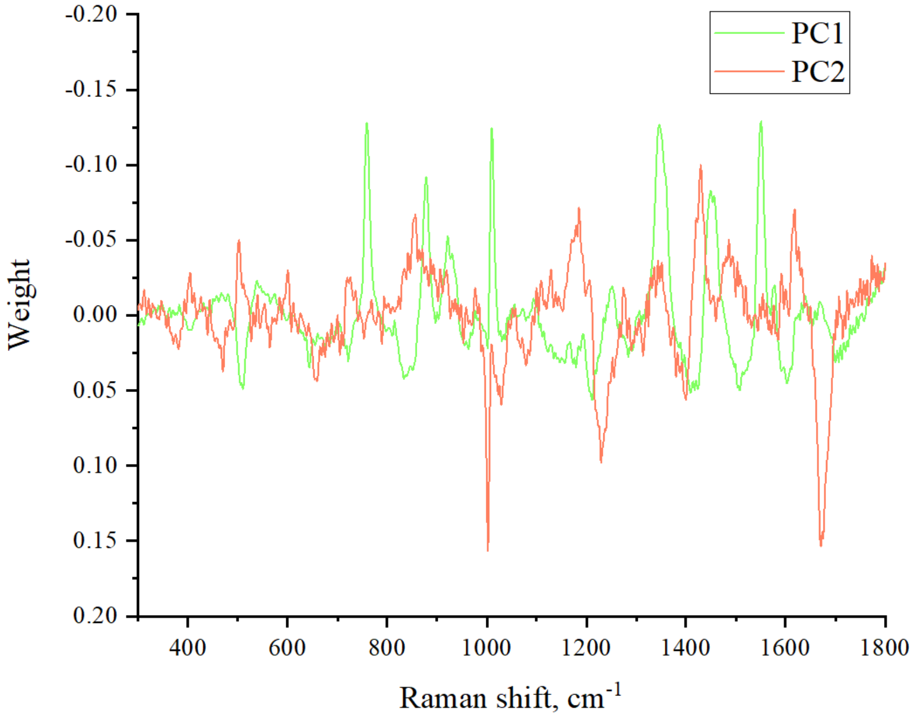

Figure S1. Loading plot (loadings spectra for the PCA results in Figure 6). The plot shows the weights for each frequency (Raman shift), that is, the influence of different frequencies on the values of the principal components (PC1 and PC2). As can be seen, various regions (marker bands), characterizing both vibrations of protein backbone and side-chains, contribute to the distinguishing of various toxins.

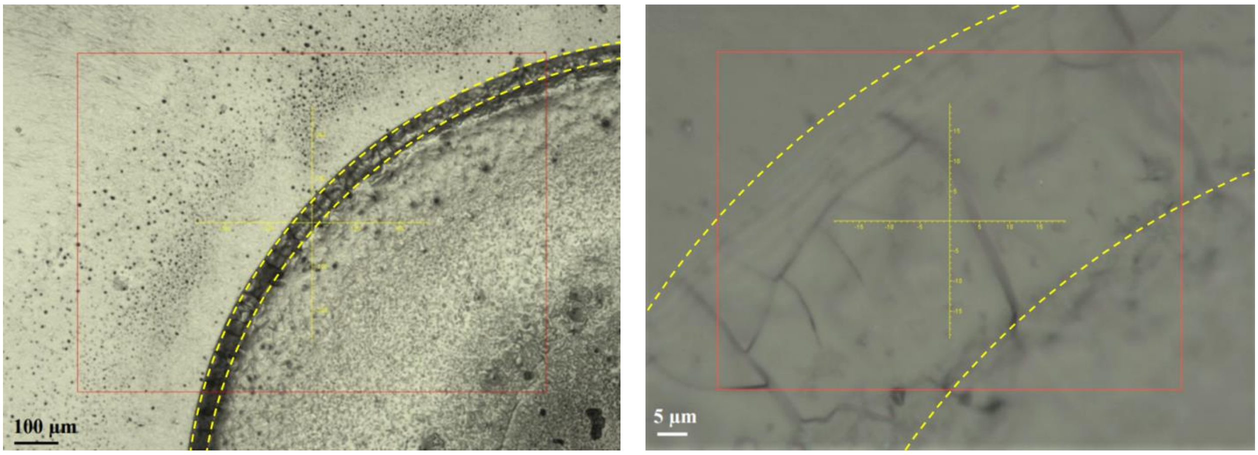

(a) (b)

Figure S2. View of the dried 2.5 µl drop of rSLURP-1 sample through a 4x (a) or 50x (b) objective lens. The crosshairs indicate the approximate location of laser radiation focusing for recording Raman spectra. Concentration of protein in the area at the droplet border (“coffee ring” effect) is demonstrated. The contours of the "ring" are approximately indicated by the yellow dotted line.
